# Combining Vibrotactile Feedback with Volitional Myoelectric Control for Robotic Transtibial Prostheses

**DOI:** 10.3389/fnbot.2016.00008

**Published:** 2016-08-22

**Authors:** Baojun Chen, Yanggang Feng, Qining Wang

**Affiliations:** ^1^The Robotics Research Group, College of Engineering, Peking University, Beijing, China

**Keywords:** vibrotactile feedback, volitional myoelectric control, human-centered closed-loop control, vibrotactile stimulation, position control, robotic transtibial prostheses

## Abstract

In recent years, the development of myoelectric control for robotic lower-limb prostheses makes it possible for amputee users to volitionally control prosthetic joints. However, the human-centered control loop is not closed due to the lack of sufficient feedback of prosthetic joint movement, and it may result in poor control performance. In this research, we propose a vibrotactile stimulation system to provide the feedback of ankle joint position, and validate the necessity of combining it with volitional myoelectric control to achieve improved control performance. The stimulation system is wearable and consists of six vibrators. Three of the vibrators are placed on the anterior side of the thigh and the other three on the posterior side of the thigh. To explore the potential of applying the proposed vibrotactile feedback system for prosthetic ankle control, eight able-bodied subjects and two transtibial amputee subjects (TT1 and TT2) were recruited in this research, and several experiments were designed to investigate subjects’ sensitivities to discrete and continuous vibration stimulations applied on the thigh. Then, we proposed a stimulation controller to produce different stimulation patterns according to current ankle angle. Amputee subjects were asked to control a virtual ankle displayed on the computer screen to reach different target ankle angles with a myoelectric controller, and control performances under different feedback conditions were compared. Experimental results indicated that subjects were more sensitive to stimulation position changes (identification accuracies were 96.39 ± 0.86, 91.11, and 93.89% for able-bodied subjects, TT1, and TT2, respectively) than stimulation amplitude changes (identification accuracies were 89.89 ± 2.40, 87.04, and 85.19% for able-bodied subjects, TT1, and TT2, respectively). Response times of able-bodied subjects, TT1, and TT2 to stimulation pattern changes were 0.47 ± 0.02 s, 0.53 s, and 0.48 s, respectively. Furthermore, for both TT1 and TT2, the absolute error of virtual ankle control reduced by about 50% with the addition of vibrotactile feedback. These results suggest that it is promising to apply the vibrotactile feedback system for the control of robotic transtibial prostheses.

## 1 Introduction

The control loop of human movement is closed by combining efferent motor output and afferent sensory feedback. For amputees, the loss of limbs not only reduces the ability of motor control but also causes the absence of some useful sensory feedback. Therefore, it is necessary to rebuild the human-centered control loop for prosthetic limbs. However, most existing studies on robotic lower-limb prostheses are focused on motor control (Sup et al., 2008; Au et al., 2009; Hitt et al., 2009; Bergelin et al., 2010; Cherelle et al., 2014; Lawson et al., 2014; Wang et al., 2015), while works on sensory feedback are limited. Though amputees could still receive some haptic feedback through the interaction between residual limbs and prosthetic sockets, the information might be insufficient for the control of a robotic prosthesis with complex functionality. In addition, the lack of sensory feedback makes it difficult for amputee users to accept prostheses as their “own limbs.” As a consequence, affording amputee users the ability to “feel” prosthetic limbs is a challenge for the development of robotic prostheses.

The goal of robotic prosthesis control is allowing amputee users to control prosthetic limbs in a natural and intuitive way, which is similar with that of controlling intact limbs. However, most existing lower-limb prostheses are controlled by their intrinsic controllers, and do not afford amputee users the freedom to directly control prosthetic joints. The control strategy is quite different from that of intact limbs, resulting in the absence of ownership feeling of prostheses. In recent years, several studies have been carried out to explore the potential of realizing volitional control of robotic lower-limb prostheses by amputee users with myoelectric controllers (Au et al., 2005; Ha et al., 2011; Dawley et al., 2013; Hoover et al., 2013; Wang et al., 2013; Huang et al., 2014; Chen et al., 2015). Ha et al. (2011) presented a volitional myoelectric controller for the control of a prosthetic knee during non-weight-bearing activity. Position control of the knee joint could be realized by estimating angular velocity of the knee joint using surface electromyographic (EMG) signals measured from the hamstring and quadriceps muscles. Hoover et al. (2013) developed a finite-state myoelectric controller for stair ascent with a powered transfemoral prosthesis. The controller combined proportional myoelectric torque control with a state-determined knee impedance to estimate knee torque using surface EMG measurements of muscles in the residual thigh. In our previous study, we designed a myoelectric controller for a robotic transtibial prosthesis (Chen et al., 2015). With the proposed controller, amputee users were able to volitionally adjust control parameters by actively contracting residual muscles in the shank, and could adaptively walk on the ground with varied slopes. These studies validated the promise of rebuilding the pathway of efferent motor output in the human-centered control loop for robotic lower-limb prostheses. However, the control loop is not closed due to the lack of sufficient feedback from prostheses, and control performance could, therefore, be limited.

Sensory substitution is an effective approach to provide feedback for prosthesis control (Antfolk et al., 2013). It transfers the feedback information through a different sensory channel or in a different modality (Kaczmarek et al., 1991). After training for a period of time, amputee users are able to understand the feedback information transferred by the sensory substitution system. There are several different sensory substitution methods, such as visual sensory substitution (Zambarbieri et al., 1998), auditory sensory substitution (Bamberg et al., 2010; Gonzalez et al., 2012; Yang et al., 2012), and tactile sensory substitution (Sabolich and Ortega, 1994; Wall and Kentala, 2005; Buma et al., 2007; Bark et al., 2008; Cipriani et al., 2008, 2012; Fan et al., 2008; Alahakone et al., 2010; Wheeler et al., 2010; Gopalai et al., 2011; Rusaw et al., 2012; Stepp et al., 2012; Witteveen et al., 2012; Erwin and Sup, 2014, 2015; Crea et al., 2015). Most visual sensory substitution systems are not portable, which makes them inconvenient to use in daily life. Auditory sensory substitution has a high requirement for quietness, and its performance may be impacted when talking with others. Therefore, auditory sensory substitution is not a satisfactory approach for daily application. Compared with sensory substitution systems based on visual or auditory feedback, tactile sensory substitution systems might be more practical for daily use, because they are usually wearable and will not disturb daily activities. Tactile feedback is usually provided through electrotactile stimulation (Sabolich and Ortega, 1994; Buma et al., 2007) or vibrotactile stimulation (Bark et al., 2008; Cipriani et al., 2008, 2012; Fan et al., 2008; Wheeler et al., 2010; Wentink et al., 2011; Rusaw et al., 2012; Stepp et al., 2012; Erwin and Sup, 2014, 2015; Crea et al., 2015). Compared with electrotactile stimulation, vibrotactile stimulation is more comfortable, which makes it easier to be accepted by amputee users (Kaczmarek et al., 1991). Vibrotactile stimulation systems usually produce different senses by changing stimulation parameters, such as stimulation position, frequency, amplitude, and duration. Different pieces of feedback information are given to users by activating corresponding stimulation patterns with specific combinations of stimulation parameters. Several studies have been carried out to explore the potential of applying vibrotactile feedback for the control of upper-limb prostheses (Bark et al., 2008; Cipriani et al., 2008, 2012; Wheeler et al., 2010; Stepp et al., 2012; Witteveen et al., 2012; Erwin and Sup, 2014, 2015). Among these studies, grasping force (Cipriani et al., 2008, 2012; Stepp et al., 2012) and joint position (Bark et al., 2008; Wheeler et al., 2010; Witteveen et al., 2012; Erwin and Sup, 2014, 2015) were two mostly used feedback information. Cipriani et al. (2012) proposed a vibrotactile feedback system to provide force feedback for an EMG controlled prosthetic hand. Grasping force of the prosthetic hand was measured by five cable tension sensors when grasping tasks were performed. To transfer the information of grasping force, vibration stimulation with a frequency proportional to the measured force was given to the user. Erwin and Sup (2015) presented a haptic feedback system for a virtual wrist prosthesis. The virtual wrist was controlled by a surface EMG-based controller, and a three-node tactor array was used to transfer the information of wrist joint position to subjects. Compared with the efforts made to develop vibrotactile feedback systems for upper-limb prostheses, limited studies were carried out for lower-limb prostheses (Fan et al., 2008; Rusaw et al., 2012; Crea et al., 2015). In these studies, useful movement information was provided to subjects through vibrotactile feedback, which helped the subjects to adjust their own body to improve walking stability. Fan et al. developed a haptic feedback system, which had four pneumatically controlled balloon actuators mounted on a cuff worn on the middle thigh. Four piezoresistive force sensors were integrated into a shoe insole to measure contact forces of four critical points of the foot. Sensory input from the foot was relayed to the leg by driving corresponding balloon actuators, and users were expected to “feel” the contact force of the foot by perceiving sequential stimuli (Fan et al., 2008). Rusaw et al. (2012) proposed a similar vibratory feedback system, which produced vibration stimulations using four tactors. The system provided transtibial prosthesis users with vibratory feedback proportional to the signal received from force transducers located under the prosthetic foot. Experimental results suggested that the use of vibratory feedback improved postural stability in transtibial prosthesis users. Unlike the two previous studies, Crea et al. (2015) presented a tactile feedback system that transferred the information of gait-phase transitions rather than the contact force of the foot to walkers. Gait-phase transitions were detected with pressure-sensitive insoles, and stimulations were produced by three vibrators placed on the thigh. When different gait-phase transitions occurred, corresponding vibrators would be activated. The feedback was expected to be helpful for gait control in lower-limb amputees. Though the above vibrotactile feedback systems could provide helpful information for lower-limb amputees to adjust their own bodies and improve walking stability, the feedback information given to amputees were used for the control of intact limbs, rather than prosthetic joints. Most of these feedback systems were tested on amputee users wearing passive prostheses, which had low requirement for prosthetic joint control. However, the feedback information might be insufficient for some robotic prostheses, whose prosthetic joints are controlled by volitional myoelectric controllers. Therefore, to close the human-centered control loop for prosthetic joints, it is necessary to rebuild the pathway of afferent sensory feedback by providing movement information of prosthetic joints to amputee users. Chew (2006) designed a vibrotactile feedback system, which embedded nine vibrator motors with the prosthetic socket liner. When a virtual ankle displayed on the computer screen was in different positions, vibrations of corresponding vibrotactile mapping patterns would be displayed. With the vibrotactile feedback, subjects were directed to control the virtual ankle to desired positions using a handled knob. Though this study explore the potential of mapping vibration patterns with ankle angle, the feedback system was not tested when working together with a myoelectric controller, which is thought to be a promising approach for amputee users to volitionally control robotic prostheses.

In this research, we aim to design a vibrotactile feedback system that feeds back the information of ankle joint position to amputee users. The system consists of six vibrators, which are placed on the anterior and posterior side of the thigh. By combining it with a volitional myoelectric controller, human-centered closed-loop control of robotic transtibial prostheses could be realized. To evaluate the promise of applying the proposed feedback system for prosthetic ankle control, eight able-bodied subjects and two transtibial amputee subjects participated in this study and four experiments were performed. The first two experiments were performed to evaluate subjects’ performance of discriminating vibrations applied on different positions or with different vibration amplitudes. The third experiment was performed to verify whether subjects were able to make fast responses to stimulation position changes when continuous vibrations were applied on the thigh. In the fourth experiment, the two amputee subjects were asked to control a virtual ankle displayed on the computer screen to reach different target ankle angles using a volitional myoelectric controller. To validate the necessity of combining vibrotactile feedback with myoelectric control, control performances of the virtual ankle under different feedback conditions (no feedback, vibrotactile feedback, and two types of visual feedback) were compared. Experimental results showed that subjects had a better performance of perceiving vibration positions than discriminating vibration amplitudes. In addition, subjects were able to perceive stimulation position changes with small time delay. Control performance of the virtual ankle with vibrotactile feedback was much better than that without any feedback, and comparable with that under visual feedback conditions. These results suggest that it is promising to apply the proposed vibrotactile feedback system for robotic transtibial prosthesis control and achieve improved control performance by combining it with volitional myoelectric controllers.

The remainder of the paper is organized as follows. We introduced the hardware for vibrotactile stimulation and EMG measurement in Section 2.1, followed by the illustration of experiment protocol in Section 2.2. In Section 3, results of four experiments were reported. The discussion was presented in Section 4, and we concluded in Section 5.

## 2 Materials and Methods

### 2.1 Hardware

#### 2.1.1 Vibrotactile Stimulation System

**Figure 1:**
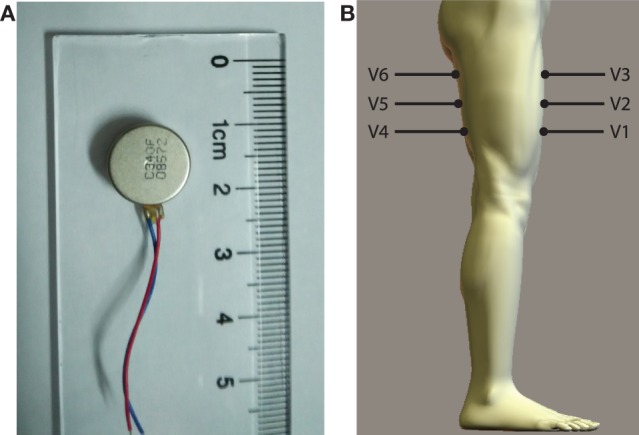
**(A)** Vibrator and **(B)** placement of vibrators (V1–V6) on the thigh.

The vibrotactile stimulation system has six miniaturized vibrators (pager motors), which are 12 mm in diameter, 3.4 mm in height, and 1.7 g in mass (Figure 1A). For this kind of vibrators, the vibration amplitude and frequency are coupled together. Therefore, only vibration amplitude is controlled in this study. Each vibrator is driven by a pulse width modulation (PWM) signal, and vibration amplitude is determined by the duty cycle of the PWM signal. In this research, the vibrators are divided into two groups: three of them (V1, V2, and V3) are placed in a line on the anterior side of the thigh, while the other three (V4, V5, and V6) on the posterior side of the thigh (Figure 1B). The distance between adjacent vibrators is about 7 cm. To improve the comfortability of wearing the stimulation system, vibrators are pasted on a thin and stretchy sleeve worn by each subject. The vibrotactile stimulation system is controlled by a self-designed driver circuit, which receives control commands sent from a host computer through a RS232 serial interface. Control commands include IDs of activated vibrators, corresponding vibration durations, and vibration amplitudes.

#### 2.1.2 EMG Measurement

**Figure 2:**
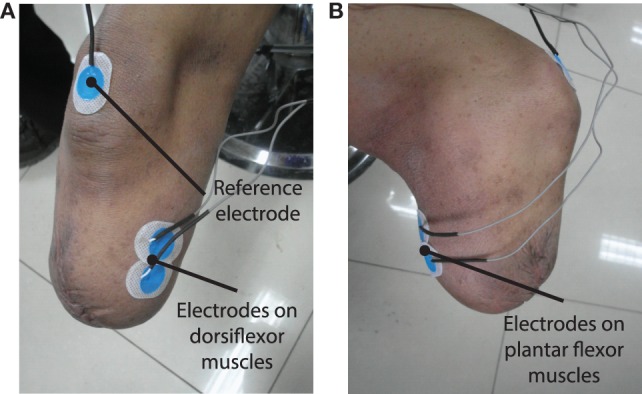
**(A,B)** show the placement of surface EMG electrodes on the residual limb to measure EMG signals from dorsiflexor and plantar flexor muscles, respectively.

Wet-gel Ag/AgCl surface electrodes (Ambu, NF-50-K) are used for EMG measurement. Two channels of EMG signals are collected from the dorsiflexor and plantar flexor muscles of amputee subject’s residual shank (Figures 2A,B), respectively. Positions for electrode placement are determined by palpation. One electrode is placed on the bony area of the knee as the reference electrode. EMG signals are differentially amplified with a gain of 1000, full-wave rectified and lower-pass filtered with a Butterworth filter, whose cutoff frequency is 2.0 Hz. Then, the signals are amplified with a gain of 10. The above signal processing is accomplished by a self-designed circuit. The processed signals are transmitted to a host computer through a data acquisition (DAQ) card (National Instruments, NI-USB-6009). The sampling rate for signal collection is 1000 Hz.

### 2.2 Subjects and Experiment Protocol

Eight able-bodied subjects and two transtibial amputee subjects (TT1 and TT2) participated in the research and provided written and informed consent. The experiment was approved by the Local Ethics Committee of Peking University (Beijing, China). Able-bodied subjects had an average age (mean ± SD) of 26.6 ± 2.7 years, height of 176.3 ± 5.4 cm, and weight of 67.6 ± 8.0 kg. TT1’s age was 34 years, height was 172 cm, and weight was 66 kg. He has been amputated (left side) for 17 years. The length of his residual shank was 12 cm (from patella to the amputated site), while the length of his sound shank was 42 cm (from patella to malleolus lateralis). TT2’s age was 27 years, height was 172 cm, and weight was 75 kg. He has been amputated (right side) for 5 years. The length of his residual shank was 22 cm, while the length of his sound shank was 43 cm.

Four experiments were performed in this study. The first three experiments were designed to evaluate the sensitivity of the thigh to different types of stimulations produced by the vibrotactile feedback system. The fourth experiment was designed to evaluate the performance of controlling a virtual ankle to reach different target positions using a myoelectric controller under different feedback conditions. Both amputee and able-bodied subjects participated in the first three experiments, while only two amputee subjects participated in the last experiment. When the four experiments were performed, subjects were asked to sit on a chair, and vibrators on the posterior side of the thigh should not contact with the seat.

#### 2.2.1 Perception of Stimulation Position Changes

The first experiment was designed to evaluate subjects’ ability to perceive the change of stimulation positions. In each trial of the experiment, subjects received two discrete vibrations sequentially. The duration of each vibration was 200 ms, the interval between them was 400 ms. These two vibrations were produced by vibrators placed at either the same position or different positions. Vibrators were activated at the maximum amplitude. Note that these two vibrations were applied on the same side (the anterior side or the posterior side) of the thigh in a single experiment trial. After these two vibrations were produced, subjects were required to judge whether these two vibrations were applied at the same position or different positions (and more specially, moving up or down), and then clicked corresponding button displayed on the computer screen. For example, if the first vibration was produced by V1 and the second vibration was produced by V2 or V3, subjects should click the button denoting “moving up”; if the first vibration was produced by V5 and the second vibration was produced by V4, subjects should click the button denoting “moving down”; if these two vibrations were produced by the same vibrator, subjects should click the button denoting “unchanged.” Button click should be completed within 3 s, otherwise it would be considered as a false identification. With the proposed vibrotactile stimulation system, 18 (3 × 3 + 3 × 3) different combinations of vibrations could be produced. In this experiment, each combination was repeated for ten times. Therefore, a total of 180 trials were tested. Test orders of experiment trials with different vibration combinations were randomly determined. Before the test trials began, subjects were asked to take several training trials. The training period terminated when subjects were familiar with the experiment task, and it usually took about 10–20 min.

#### 2.2.2 Perception of Stimulation Amplitude Changes

The second experiment was designed to evaluate subjects’ ability to perceive the change of stimulation amplitudes. Similar to experiment 1, in each trial of the experiment, subjects received two discrete vibrations sequentially. The duration of each vibration was 200 ms, and the interval between them was 400 ms. These two vibrations were produced by the same vibrator. The amplitude of each vibration could be 0, 50, or 100% maximum amplitude. Each experiment trial had three periods: the first stimulation period, the interval, and the second stimulation period. To avoid mistaking vibrations with 0% maximum amplitude (i.e., no vibration was produced) happened in the first stimulation period as the second stimulation, the current experiment period (the first stimulation, the interval, or the second stimulation) was displayed on the computer screen. After the two stimulations were produced, subjects should judge whether stimulation amplitude increased, unchanged or decreased (the second stimulation was compared with the first one), and then clicked corresponding button displayed on the computer screen. The button should be clicked within 3 s, otherwise this identification would be considered as a false one. For each vibrator, there were 9 (3 × 3) different combinations of vibrations. In this study, all the stimulation combinations were tested for all the six vibrators and repeated for three times. Therefore, a total of 162 test trials were taken. The test order of experiment trials with different vibration combinations was randomly determined. Before test trials began, subjects took several training trials to get familiar with the experiment task.

#### 2.2.3 Response to Stimulation Position Changes

The third experiment was designed to evaluate subjects’ ability to make fast responses when the stimulation position changed. In each trial of the experiment, a continuous stimulation sequence with vibration position changes was applied on the subjects. For each stimulation sequence, a row of three vibrators on the anterior side or posterior side of the thigh were activated sequentially from up to down or from down to up. Vibration amplitude of each vibrator was set to be the maximum amplitude. In this research, four kinds of stimulation sequences were tested: V1 → V2 → V3, V3 → V2 → V1, V4 → V5 → V6, and V6 → V5 → V4. To avoid subjects predicting the moment of stimulation position changes, vibration duration of each vibrator was randomly ranged from 1 to 2 s. When the activation of one vibrator was terminated, the next vibrator would be activated immediately. Subjects should click a button displayed on the computer screen as soon as possible after they perceived the change of vibration position or the beginning of the stimulation sequence. As a consequence, the button should be clicked for three times in each experiment trial. In this study, each stimulation sequence was tested for 15 times and, therefore, a total of 60 test trials were taken. The test order of experiment trials with different stimulation sequences was randomly determined. Between two adjacent test trials, 5-s rest was allowed. To quantitatively evaluate the performance of each subject, response time (*T_R_*) of perceiving stimulation position changes is calculated by

(1) $$T_{R}=t_{C}-t_{S},$$

where *t_C_* denotes the moment of button click and *t_S_* denotes the moment of stimulation position changes.

#### 2.2.4 Virtual Ankle Control

**Figure 3:**
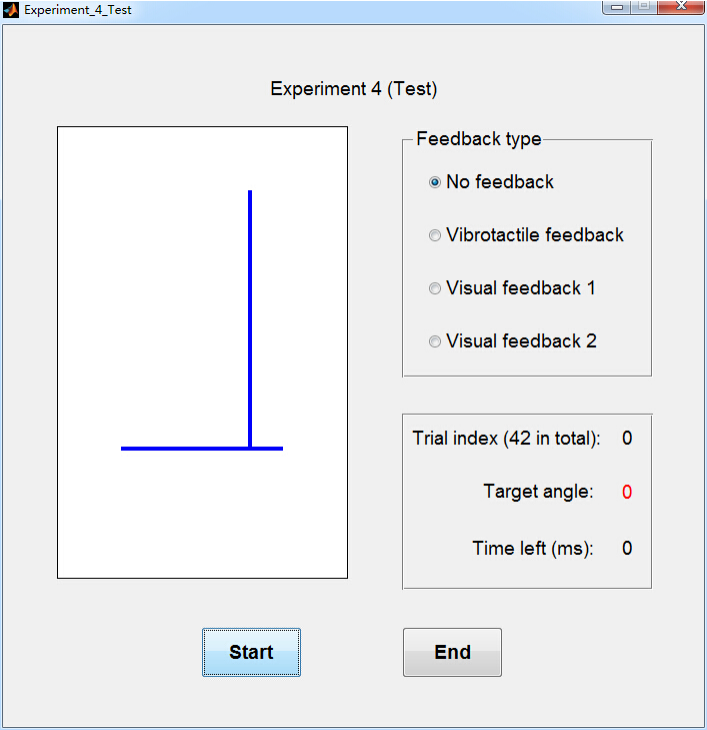
**Graphic user interface (GUI) for experiment 4**. The left graph displays the virtual ankle. Top right part of the GUI shows the selected feedback type and bottom right part of the GUI shows the index and target angle of current experiment trial, as well as the time left for virtual ankle control.

The fourth experiment was designed to validate the necessity of combining vibrotactile feedback with volitional myoelectric control. In this experiment, amputee subjects were asked to control a virtual ankle displayed on the computer screen to reach target ankle angles under four different feedback conditions (Figure 3).

The experiment task is similar to that performed in our previous study (Chen et al., 2014). With a previously designed myoelectric controller [please refer to Chen et al. (2014) for more details], amputee subjects were able to volitionally control the virtual ankle by actively contracting residual plantar flexor muscles and dorsiflexor muscles. To train the myoelectric control model, subjects were asked to consciously perform dorsiflexion and plantar flexion of the “phantom” ankle with different muscle contraction intensities. EMG signals were collected and processed using 10-ms adjacent sliding windows. Average amplitudes were calculated for the two channels of EMG signals measured by the circuit, and then they were normalized by dividing the maximum amplitudes of the two channels, respectively. The normalized data were mapped to the joint angle of the virtual ankle with the myoelectric control model. Position of the ankle joint was updated every 10 ms. Myoelectric controller was trained before the experiment, and it took about 10 min.

Movement range of the virtual ankle is from −17.5° to 17.5°, where positive value denotes dorsiflexion and negative value denotes plantar flexion. In this experiment, 7 target ankle angles were tested: 0°, ±5°, ±10°, and ±15°. Each target position was tested for 6 times and, therefore, a total of 42 test trials were taken. The test order of experiment trials with different target ankle angles was randomly determined. Before each test trial began, there was a 5-s preparation period and the value of target angle was displayed on the computer screen for subjects to be prepared. When the test trials began, only 1 s was left for subjects to control the virtual ankle to reach target ankle angles, and average angle of the last 200 ms was calculated for the evaluation of control performance. The test trial was designed to mimic the scenario of the swing period during walking. In this period of a gait cycle, position control of the ankle joint plays an important role in preventing the foot from dragging along the ground and absorbing shocks when the foot strikes on the ground.

The above test trials were performed under four feedback conditions: no feedback, visual feedback 1, visual feedback 2, and vibrotactile feedback. Absolute errors of virtual ankle control under these four feedback conditions were compared. In “no feedback” condition, no feedback was given to amputee subjects when they controlled the virtual ankle. In “visual feedback 1” condition, the movement of the virtual ankle was displayed on the computer screen, but the target position was not marked. In “visual feedback 2” condition, the movement of the virtual ankle was displayed on the computer screen, and the target position was also marked. In “vibrotactile feedback” condition, subjects received vibration stimulations produced by different vibrators in real time. The ID of activated vibrator was determined by current joint angle of the virtual ankle (Table 1). Experiment trials of virtual ankle control with vibrotactile feedback were performed as follows. At the beginning of each trial, amputee subjects relaxed their residual muscles and no vibrator was activated (ankle angle should be about 0°). When subjects volitionally control the virtual ankle to different positions, corresponding vibrators would be activated. By perceiving the change of vibration position, subjects could be aware of whether to increase or decrease the intensity of residual muscle contraction.

**Table 1:** **Vibration patterns for different ankle angle ranges**.

Ankle angle range (deg)	Activated vibrator
−17.5 to −12.5	V6
−12.5 to −7.5	V5
−7.5 to −2.5	V4
−2.5 to +2.5	None
+2.5 to +7.5	V1
+7.5 to +12.5	V2
+12.5 to +17.5	V3

## 3 Results

### 3.1 Perception of Stimulation Position Changes

The average identification accuracy of perceiving stimulation position changes over eight able-bodied subjects was 96.39 ± 0.86% (i.e., mean ± SEM). The performance of discriminating vibrations applied on the anterior side of the thigh (97.36 ± 0.73%) was a little higher than that of the posterior side (95.42 ± 1.20%). To determine whether the difference was statistically significant, a paired-samples *t*-test was performed. There were no outliers in the data, as assessed by inspection of a boxplot. The assumption of normality was not violated, as assessed by Shapiro–Wilk’s test (*p* = 0.170). Result of paired-samples *t*-test revealed that accuracies of discriminating vibrations applied on the anterior side and posterior side of the thigh showed no statistically significant difference (*p* = 0.087). To make a further understanding of how identification errors distributed, we calculated the identification accuracy for each vibration combination (Table 2). For vibrations applied on the anterior side of the thigh, most of the errors happened when V1 → V2 and V2 → V1 were performed. For vibrations applied on the posterior side of the thigh, most of the errors happened when V4 → V5, V5 → V4, V5 → V6, and V6 → V5 were performed.

**Table 2:** **Average identification accuracies (mean ± SEM) (%) over eight able-bodied subjects for different combinations of vibrator activation**.

First stimulation	Second stimulation		
	V1	V2	V3
**V1**	100.00 ± 0.00	95.00 ± 1.89	97.50 ± 2.50
**V2**	96.25 ± 2.63	95.00 ± 3.78	98.75 ± 1.25
**V3**	100.00 ± 0.00	97.50 ± 1.64	96.25 ± 2.63

	**V4**	**V5**	**V6**

**V4**	96.25 ± 1.83	93.75 ± 2.63	100.00 ± 0.00
**V5**	93.75 ± 2.63	97.50 ± 1.64	93.75 ± 3.24
**V6**	98.75 ± 1.25	87.50 ± 6.20	97.50 ± 1.64

*The top table shows the result of discriminating vibrations applied on the anterior side of the thigh, while the bottom table shows the result of discriminating vibrations applied on the posterior side of the thigh*.

Identification accuracies of TT1 and TT2 were 91.11 and 93.89%, respectively. Similar to the result of able-bodied subjects, TT1 had better identification performance when vibrations were applied on the anterior side (92.22%) than applied on the posterior side (90.00%) of the thigh. Most of the errors happened when V2 → V3 and V3 → V2 were performed on the anterior side, and V5 → V4, V5 → V6, and V6 → V5 performed on the posterior side (Table 3). For TT2, the performance of discriminating vibrations applied on the posterior side (96.67%) was better than that applied on the anterior side (91.11%), and most of the errors were caused by the misidentification of V2 → V3 (Table 4).

**Table 3:** **Identify accuracies (%) of TT1 for different combinations of vibrator activation**.

First stimulation	Second stimulation		
	V1	V2	V3
**V1**	100.00	100.00	100.00
**V2**	90.00	90.00	70.00
**V3**	100.00	80.00	100.00

	**V4**	**V5**	**V6**

**V4**	100.00	90.00	100.00
**V5**	80.00	90.00	80.00
**V6**	90.00	80.00	100.00

*The top table shows the result of discriminating vibrations applied on the anterior side of the thigh, while the bottom table shows the result of discriminating vibrations applied on the posterior side of the thigh*.

**Table 4:** **Identify accuracies (%) of TT2 for different combinations of vibrator activation**.

First stimulation	Second stimulation		
	V1	V2	V3
**V1**	100.00	90.00	100.00
**V2**	90.00	100.00	60.00
**V3**	90.00	90.00	100.00

	**V4**	**V5**	**V6**

**V4**	100.00	100.00	100.00
**V5**	90.00	90.00	90.00
**V6**	100.00	100.00	100.00

*The top table shows the result of discriminating vibrations applied on the anterior side of the thigh, while the bottom table shows the result of discriminating vibrations applied on the posterior side of the thigh*.

### 3.2 Perception of Stimulation Amplitude Changes

**Figure 4:**
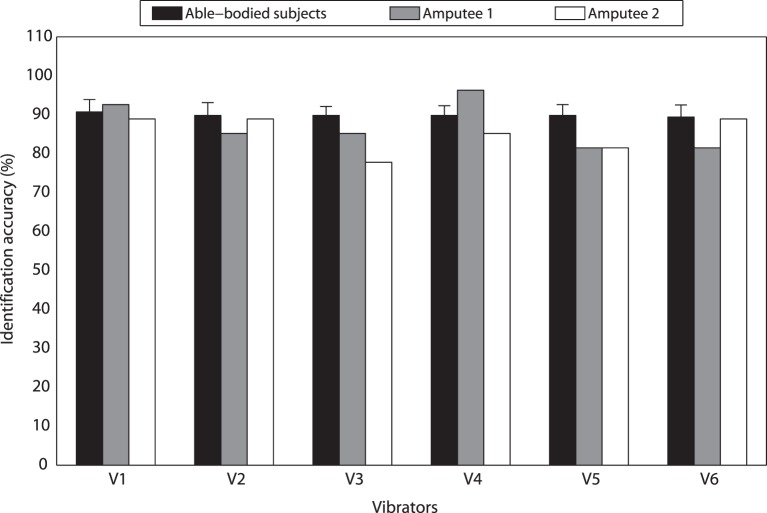
**Identification accuracies of discriminating vibration amplitude changes for different vibrators**.

**Figure 5:**
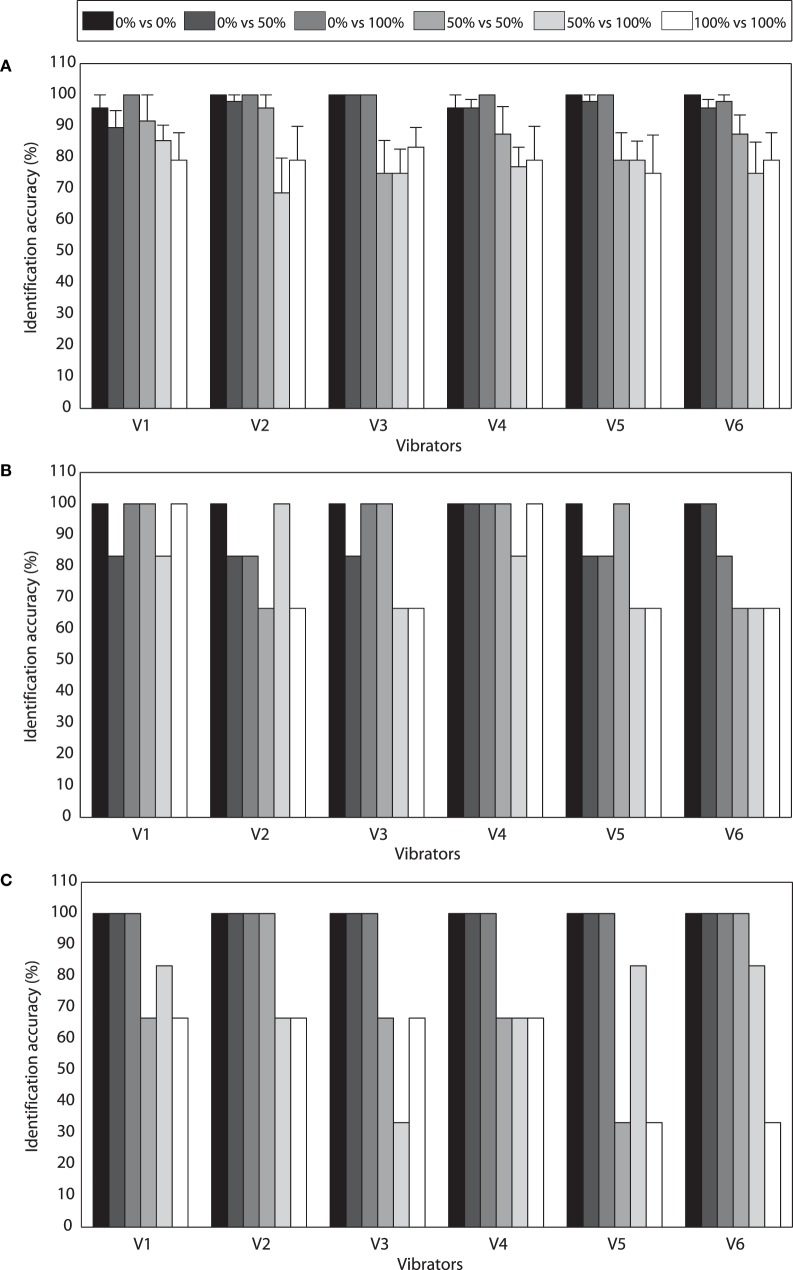
**(A)** Average identification accuracies (mean ± SEM) (%) over eight able-bodied subjects for different combinations of vibration amplitude and different vibrators. **(B)** Identification accuracies of TT1 for different combinations of vibration amplitude and different vibrators. **(C)** Identification accuracies of TT2 for different combinations of vibration amplitude and different vibrators.

The overall identification accuracy of perceiving stimulation amplitude changes over eight able-bodied subjects was 89.89 ± 2.40%. For each individual vibrator V1, V2, V3, V4, V5, and V6, average accuracies over eight able-bodied subjects were 90.74 ± 3.21, 89.81 ± 3.34, 89.81 ± 2.29, 89.81 ± 2.50, 89.81 ± 2.78, and 89.35 ± 3.09%, respectively (Figure 4). To make a further understanding of how amplitude combinations influenced the identification performance, we calculated average identification accuracies over the six vibrators for 0 vs. 0%, 0 vs. 50% (including 0 → 50% and 50 → 0%), 0 vs. 100% (including 0 → 100% and 100 → 0%), 50 vs. 50%, 50 vs. 100% (including 50 → 100% and 100 → 50%), and 100 vs. 100% maximum amplitude, respectively (Figure 5A). Corresponding accuracies of these stimulation combinations were 98.61 ± 1.39, 96.18 ± 1.48, 99.65 ± 0.35, 86.11 ± 5.14, 76.74 ± 5.96, and 79.17 ± 7.70%, respectively. A two-way repeated measures ANOVA was performed to determine the effects of different vibration positions and vibration amplitude combinations on identification performance. There were no outliers, as assessed by examination of studentized residuals for values greater than ±3. There was no statistically significant interaction between vibration position and vibration amplitude (*p* = 0.440). The main effect of vibration position showed no statistically significant difference in identification accuracy (*p* = 0.975). But the main effect of vibration amplitude combination showed that there was a statistically significant difference in identification accuracy (*p* < 0.01). *Post hoc* pair-wise comparisons showed that identification performance of the first three stimulation combinations were significantly better than those of the last three combinations (*p* < 0.05 for all pair-wise comparisons, i.e., any combination from the first three was compared with any combination from the last three). In addition, all pair-wise comparisons among the first three combinations and those among the last three combinations were not statistically significant.

Experimental results of TT1 and TT2 were similar with those of able-bodied subjects. For TT1, identification accuracies of vibrations applied on V1, V2, V3, V4, V5, and V6 were 92.59, 85.19, 85.19, 96.30, 81.48, and 81.48%, respectively (Figure 4). Average accuracies over the six vibrators were 100.00, 88.89, 91.67, 88.89, 77.78, and 77.78% for 0 vs. 0%, 0 vs. 50%, 0 vs. 100%, 50 vs. 50%, 50 vs. 100%, and 100 vs. 100% maximum amplitude, respectively (Figure 5B). For TT2, identification accuracies of vibrations applied on V1, V2, V3, V4, V5, and V6 were 88.89, 88.89, 77.78, 85.19, 81.48, and 88.89%, respectively (Figure 4). Average accuracies over the six vibrators were 100.00, 100.00, 100.00, 72.22, 69.44, and 55.56% for 0 vs. 0%, 0 vs. 50%, 0 vs. 100%, 50 vs. 50%, 50 vs. 100%, and 100 vs. 100% maximum amplitude, respectively (Figure 5C). For both able-bodied and amputee subjects, most of the errors were caused by the misidentification of 50 vs. 50%, 50 vs. 100%, and 100 vs. 100% maximum amplitude. The results indicate that it is more difficult for subjects to discriminate the amplitude of a vibration (50 and 100% maximum amplitude) than judge whether a vibration happens.

We also performed a paired-samples *t*-test to compare identification performance of vibration position changes and vibration amplitude changes. No outliers were found in the data, as assessed by inspection of a boxplot. The assumption of normality was not violated, as assessed by Shapiro–Wilk’s test (*p* = 0.508). Compared with the performance of discriminating stimulation amplitude changes, identification accuracy of discriminating stimulation position changes had an increment of 6.50 ± 5.73%, and the difference was statistically significant (*p* = 0.015).

### 3.3 Response to Stimulation Position Changes

**Figure 6:**
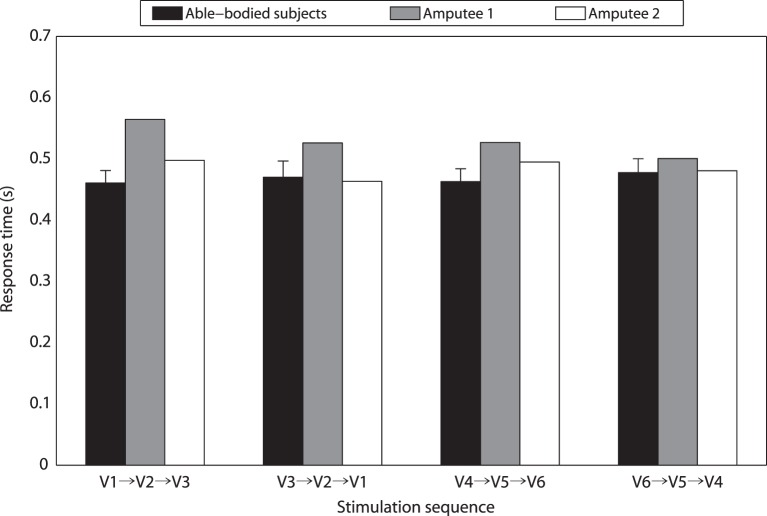
**Response times for different stimulation sequences**.

The average response time to stimulation position changes was 0.47 ± 0.02 s over eight able-bodied subjects. For different vibration sequences, average response times to V1 → V2 → V3, V3 → V2 → V1, V4 → V5 → V6, and V6 → V5 → V4 were 0.46 ± 0.02, 0.47 ± 0.03, 0.46 ± 0.02, and 0.48 ± 0.02 s, respectively (Figure 6). To determine whether the difference was statistically significant, a one-way repeated measures ANOVA was performed. There were no outliers in the data, as assessed by inspection of a boxplot. Response time was normally distributed for each vibration sequence, as assessed by Shapiro–Wilk’s test (*p* > 0.05). Mauchly’s test of sphericity showed that the assumption of sphericity had not been violated (*p* = 0.603). Response times to different vibration sequences showed no statistically significant difference (*p* = 0.212). The overall response time of TT1 and TT2 were 0.53 and 0.48 s, respectively. Response times of TT1 were 0.56, 0.53, 0.53, and 0.50 s, respectively, for V1 → V2 → V3, V3 → V2 → V1, V4 → V5 → V6, and V6 → V5 → V4. As for TT2, corresponding response times were 0.50, 0.46, 0.49, and 0.48 s, respectively. The results indicated that the average response time of amputee subjects was a little longer than that of able-bodied subjects. In addition, the four types of stimulation sequences did not make a significant difference to response performance.

### 3.4 Virtual Ankle Control

**Figure 7:**
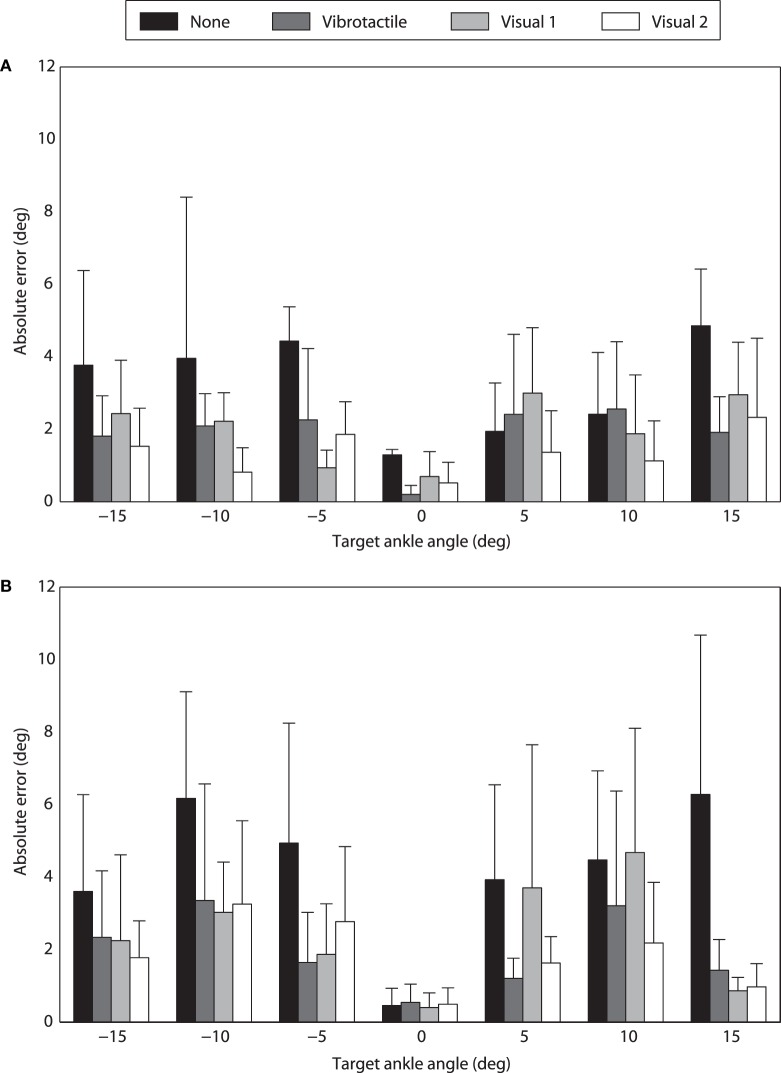
**Performance of virtual ankle control under different feedback conditions**. **(A)** Absolute errors of TT1 for different target ankle angles. **(B)** Absolute errors of TT2 for different target ankle angles.

For TT1, the overall absolute errors of virtual ankle control under different feedback conditions (i.e., no feedback, vibrotactile feedback, visual feedback 1, and visual feedback 2) were 4.38°, 2.18°, 2.49°, and 1.88°, respectively (Figure 7A). For different target ankle angles, control performances under different feedback conditions were not exactly the same. When the target ankle angle was −15°, −10°, −5°, 0° or +15°, TT1 produced much larger errors for virtual ankle control under no feedback condition than the other feedback conditions. When the target ankle angle was +5° or +10°, control performances under no feedback, vibrotactile feedback, and visual feedback 1 were close to each other, but a little worse than that under visual feedback 2 condition. For target ankle angles of −15°, −10°, −5°, 0°, +5°, +10°, and +15°, average control errors over the four feedback conditions were 2.38°, 2.27°, 2.37°, 0.68°, 2.18°, 2.00°, and 3.01°, respectively. Though the control error was small for 0° target angle, it was not 0. It is probably caused by the variation of EMG signals and external signal noise. Control performance of TT2 was similar with that of TT1. The overall absolute errors of virtual ankle control under the four feedback conditions were 4.79°, 2.45°, 2.43°, and 1.91°, respectively (Figure 7B). Average control errors over the four feedback conditions were 2.49°, 3.95°, 2.80°, 0.47°, 2.61°, 3.63°, and 2.38°, respectively, for target ankle angles of −15°, −10°, −5°, 0°, +5°, +10°, and +15°. For most of the target angles, the largest control error was produced under no feedback condition, which was in consistence with the overall performance. The experimental results indicate that the overall absolute error of virtual ankle control greatly reduced (by about 50%) when any types of feedback (vibrotactile or visual) was given. The overall control performance under vibrotactile feedback condition was similar to that under visual feedback 1 condition, and only 0.30° and 0.54° larger than that under visual feedback 2 condition for TT1 and TT2, respectively.

## 4 Discussion

The long-term goal of robotic prosthesis control is allowing amputee users to control prosthetic limbs as their “own limbs.” Compared with traditional prosthetic controllers, human-centered control is more similar with the control of intact limbs. It allows amputee users to play a more important role in the control loop of robotic prostheses, and makes it more effective to coordinate with the movement of intact limbs and prosthetic limbs. However, most existing human-centered controllers for robotic lower-limb prostheses are open-loop. Though amputee users are able to directly control prosthetic joints with volitional myoelectric controllers, they receive insufficient feedback from robotic prostheses, which could limit the control performance. To close the loop of human-centered control, it is necessary to add artificial feedback of prosthetic joint movement to existing control systems. Therefore, we designed a vibrotactile feedback system in this research, and performed several experiments to evaluate the promise of applying it for the control of robotic lower-limb prostheses. Though some vibrotactile feedback systems have been developed for lower-limb prostheses in existing studies, most of them focused on providing feedback information (e.g., contact force of the foot or moments of specific gait events) for the adjustment of intact limbs rather to improve walking stability. Compared with these studies, the aim of this study is to improve the performance of prosthetic joint control with a volitional myoelectric controller. To achieve this goal, a vibrotactile feedback system is combined with volitional myoelectric control to close the human-centered control loop, which could improve the intuitiveness of human–machine interaction.

To make the vibrotactile feedback system more practical for prosthesis control, its design should follow three rules. First, vibrations produced by the stimulation system should be easily perceived, and different stimulation patterns should be correctly discriminated by amputee users. In this study, subjects were found to be more sensitive to stimulation position changes than stimulation amplitude changes. To improve the performance of discriminating different stimulation patterns, only vibration positions were changed for different stimulation patterns, while the vibration amplitude was set to be the maximum amplitude. Second, the provided feedback information should be helpful for the current prosthetic control system. In this study, we aim to propose a human-centered closed-loop controller for prosthetic ankle. Therefore, feedback of ankle joint position might be more appropriate than other types of feedback (e.g., contact force of the foot). In addition, accurate control of ankle joint position is important for robotic transtibial prostheses, as it is helpful to avoid the foot dragging along the ground during swing phase and improve the adaptability of walking on uneven terrains. Third, the mapping relationships between vibration patterns and feedback information should be easy to learn, allowing amputee users to understand the transferred feedback with low cognitive burden. As dorsiflexion and plantar flexion are movements in the sagittal plane, according to our experience, it is easier to map the feedback information with vibrations applied on the anterior/posterior side than on the medial/lateral side of the thigh. Therefore, three of the vibrators were placed on the anterior side of the thigh, and the other three on the posterior side, although the medial side of the thigh was found to be more sensitive to vibrotactile stimulations than the anterior side in a previous study (Wentink et al., 2011).

In this study, seven different vibration patterns were defined: one pattern corresponds to no vibration and the other six patterns correspond to vibrations produced by six individual vibrators. If more vibrators were used in the vibrotactile feedback system, more vibration patterns could be defined, and the resolution of the feedback information would be improved. However, it is easier to cause confusion of vibrations produced by adjacent vibrators with closer distance, as most of the identification errors found in the first experiment were caused by the confusion of vibrations produced by adjacent vibrators. Due to space limitation of the thigh for vibrator placement, only three vibrators were placed on each side according to our experience. Though the range of virtual ankle movement is divided into seven segments, the resolution of the feedback information could be improved using some skills. For example, if the stimulated position is kept at the lower part of the thigh in the anterior side, current angle of virtual ankle is around +5°; if the stimulated position is varied between the lower part and middle part of the thigh in the anterior side, current angle of virtual ankle is around +7.5°.

Combining the proposed vibrotactile feedback system with volitional myoelectric control is promising for improving the performance of prosthetic ankle control. For both TT1 and TT2, compared with the performance of virtual ankle control without any feedback, the performance greatly improved by about 50% when vibrotactile feedback was provided. Furthermore, control performance with vibrotactile feedback was comparable to that with visual feedback 1, and only a little worse (the average absolute error over two amputee subjects increased by 0.42°) than that with visual feedback 2. However, unlike robotic hands, visual feedback is unpractical for robotic leg control, as amputee users cannot always looking down at their feet during walking. By contrast, vibrotactile feedback is more appropriate for the control of robotic lower-limb prostheses, as it will not cause any obvious inconvenience to amputee users during walking.

**Figure 8:**
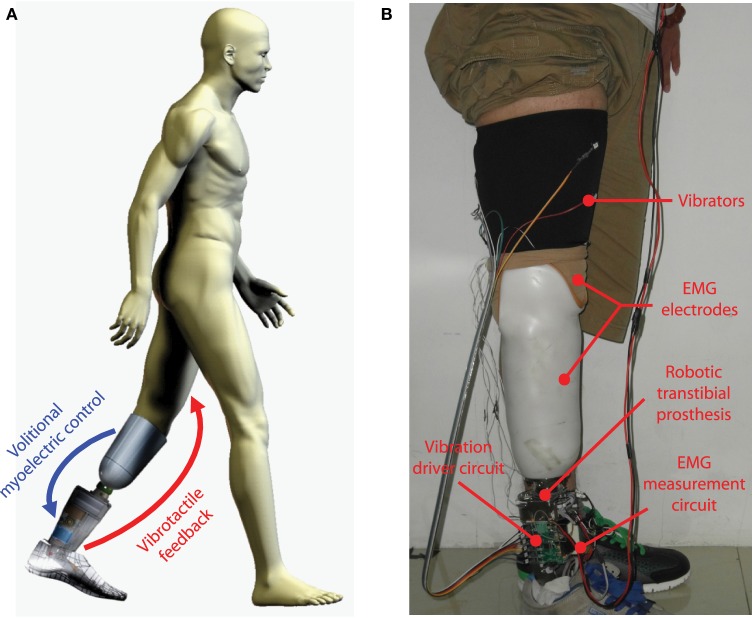
**(A)** Concept of closing the human-centered control loop for robotic transtibial prosthesis control. **(B)** The wearing of a robotic prosthesis (integrated with the systems of volitional myoelectric control and vibrotactile feedback) by a transtibial amputee subject.

Though experimental results in this research are promising, there are still some works to do to further validate the viability of applying the vibrotactile feedback system for robotic transtibial prosthesis control. In current study, the proposed vibrotactile feedback system was only tested when subjects were seated. Whether amputee subjects could still achieve satisfactory performance of perceiving vibrations and discriminating different stimulation patterns when walking with prostheses is unknown. Compared with seated experiment trials, amputee users will receive more tactile interference when walking with prostheses, which might cause the reduction of sensitivity to vibrotactile stimulation. In addition, the requirement for response time to stimulation position changes could also increase, especially when walking at a fast speed. As a consequence, to satisfy the above requirement, more training and better stimulation techniques might be necessary. In our future work, we aim to integrate the vibrotactile stimulation system and myoelectric controller with a robotic transtibial prosthesis to close the human-centered control loop (Figure 8A). When amputee users walk with robotic transtibial prostheses, they are able to volitionally control the joint angle of prosthetic ankle with the myoelectric controller during swing phase. Meanwhile, they will receive vibrotactile stimulations corresponding with current ankle angle. In this case, closed-loop control of prosthetic ankle can be realized, and control performance of ankle angle during swing phase could, therefore, be improved. The wearing of vibrotactile feedback system, volitional myoelectric control system and a robotic prosthesis [adapted from our previous prosthesis PKU-RoboTPro (Wang et al., 2015)] by a transtibial subject is shown in Figure 8B. We will test whether the human-centered closed-loop controller could improve the performance of adaptively walking on the ground with varied slopes, and it is a follow-up of our previous study (Chen et al., 2015).

## 5 Conclusion

In this study, we propose a vibrotactile stimulation system to provide feedback of ankle joint position, and explore the potential of combining it with volitional myoelectric control to close the human-centered control loop for robotic transtibial prostheses. By activating vibrators placed on different positions of the thigh, the presented vibrotactile feedback system makes it easy for users to perceive different vibration patterns and understand the ankle angle transferred by the stimulation. Experimental results of virtual ankle control on two transtibial amputees suggest that it could be helpful to add vibrotactile feedback to the control loop, and it is promising to achieve improved control performance of robotic transtibial prostheses.

## Author Contributions

QW and BC designed research; BC, YF, and QW performed research; BC analyzed data; and BC and QW wrote the paper.

## Conflict of Interest Statement

The authors declare that the research was conducted in the absence of any commercial or financial relationships that could be construed as a potential conflict of interest.
